# Case report: An uncommon presentation of extramedullary plasmacytoma without a concurrent diagnosis of multiple myeloma

**DOI:** 10.3389/fonc.2024.1353943

**Published:** 2024-06-07

**Authors:** Katarzyna Stawarz, Adam Galazka, Anna Gorzelnik, Monika Durzynska, Karolina Bienkowska-Pluta, Jakub Zwolinski

**Affiliations:** ^1^ Head and Neck Cancer Department, Maria Sklodowska-Curie National Research Institute of Oncology, Warsaw, Poland; ^2^ Department of Pathology, Maria Sklodowska-Curie, National Research Institute of Oncology, Warsaw, Poland

**Keywords:** extramedullary plasmacytoma, multiple myeloma, case report, radiotherapy, surgery

## Abstract

**Introduction:**

Extramedullary plasmacytoma (EMP) is an uncommon solitary tumor originating from neoplastic plasma cells located outside the bone marrow. Despite its rarity, the occurrence of EMP without a concurrent diagnosis of multiple myeloma (MM) is considered extremely rare. Approximately 80–90% of EMP cases are found in the head and neck region, with a higher incidence in men aged between 50 and 60 years. The current treatment modalities include radiotherapy (RT) as a first-line approach, with surgery or chemotherapy regarded as other therapeutic options. While RT proves effective in the majority of EMP cases, there are instances where the tumor remains refractory to radiation. In this case report, we present an unusual scenario of EMP resistant to RT without concurrent signs of multiple myeloma which was successfully treated with surgery followed by systemic therapy.

**Case report:**

A 72-year-old male was admitted to the Head and Neck Cancer Clinic with a 6-month history of swallowing difficulties. He denied experiencing weight loss or pain on swallowing. Basic laboratory tests yielded results within normal limits, except for beta-2 microglobulin. Physical examination revealed an enlarged submandibular lymph node on the right side. Fiberoptic examination identified a soft tissue polypoid mass within the right piriform fossa, slightly protruding into the vocal slit. A CT scan displayed a well-circumscribed 2 cm polypoid, homogeneously enhancing soft tissue mass adjacent to the posterior surface of the epiglottis and the right side of the tongue base. Bone marrow biopsy revealed no abnormalities, and there were no clinical or laboratory signs of multiple myeloma. Based on the tumor biopsy results and imaging studies, a diagnosis of EMP was made. Due to the lack of response to RT, surgical removal of the tumor was pursued, followed by systemic therapy. Ultimately, the patient achieved full recovery with effective disease control.

**Conclusion:**

In conclusion, EMP without concurrent multiple myeloma is an exceedingly rare condition that demands a multidisciplinary approach for both diagnosis and treatment. Moreover, although RT continues to be the primary standard treatment for EMP, in some cases other therapeutic regimens prove to be successful.

## Introduction

Extramedullary plasmacytoma (EMP) is a rare solitary tumor originating from neoplastic plasma cells located outside the bone marrow. It does not involve the bone marrow and can manifest anywhere in the body, with a predilection for soft tissues, particularly in the head and neck region (1, 2). EMP accounts for approximately one-third of solitary plasmacytoma tumors, constituting up to 5% of all plasma cell malignancies (3). The upper respiratory and digestive tracts are the most common sites for EMP, representing 80–90% of cases (4). This slow-growing submucosal malignancy typically presents its initial symptoms related to the mass effect, resulting in the obstruction of local structures and causing dysphagia, odynophagia, or voice problems. Male patients are more frequently affected, with an average age at diagnosis in their fifth to sixth decade of life (5). While EMP is regarded as an uncommon malignancy which can sometimes progresses to multiple myeloma, the occurrence of EMP without multiple myeloma remain exceedingly rare (6). The diagnosis of EMP is established through tumor and bone marrow biopsies, as well as laboratory tests and imaging studies (7). Although radiotherapy (RT) is the currently recommended treatment option due to the tumor’s radiosensitivity and excellent disease control, its efficacy may be insufficient in certain cases (8). Moreover, the challenging location of the tumor underscores the importance of individualizing the optimal therapeutic approach. In instances of resistance to RT, surgery and chemotherapy remain viable therapeutic alternatives that have proven effective in some patients (9). In this case scenario we described a patient with the EMP resistant to RT with no signs of multiple myeloma which was successfully treated with surgery and systemic therapy.

## Case report

**Figure 1:**
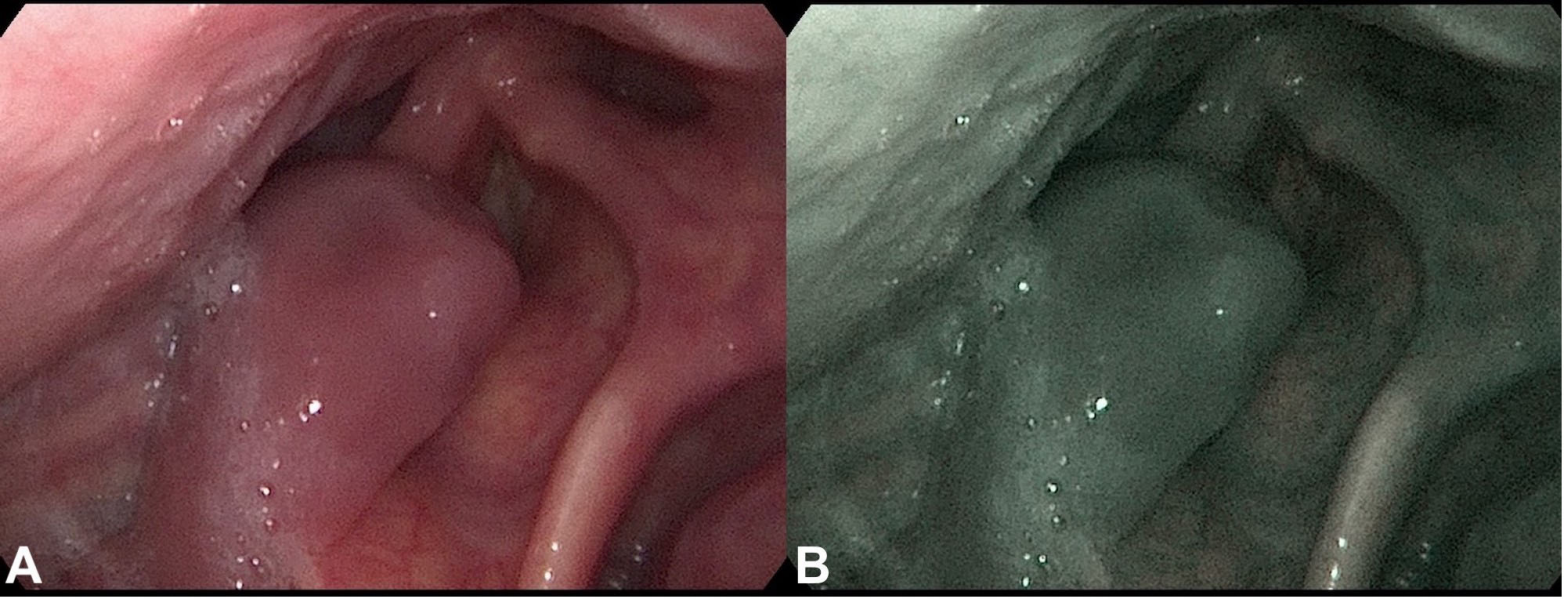
Fiberoptic examination of the throat tumor. Fiberoptic exam displaying a polypoid throat protruding from the right piriform fossa, partially occluding the vocal slit during phonation. **(A)** Conventional white-light image. **(B)** NBI (Narrow Band Imaging) image of the same site.

**Figure 2:**
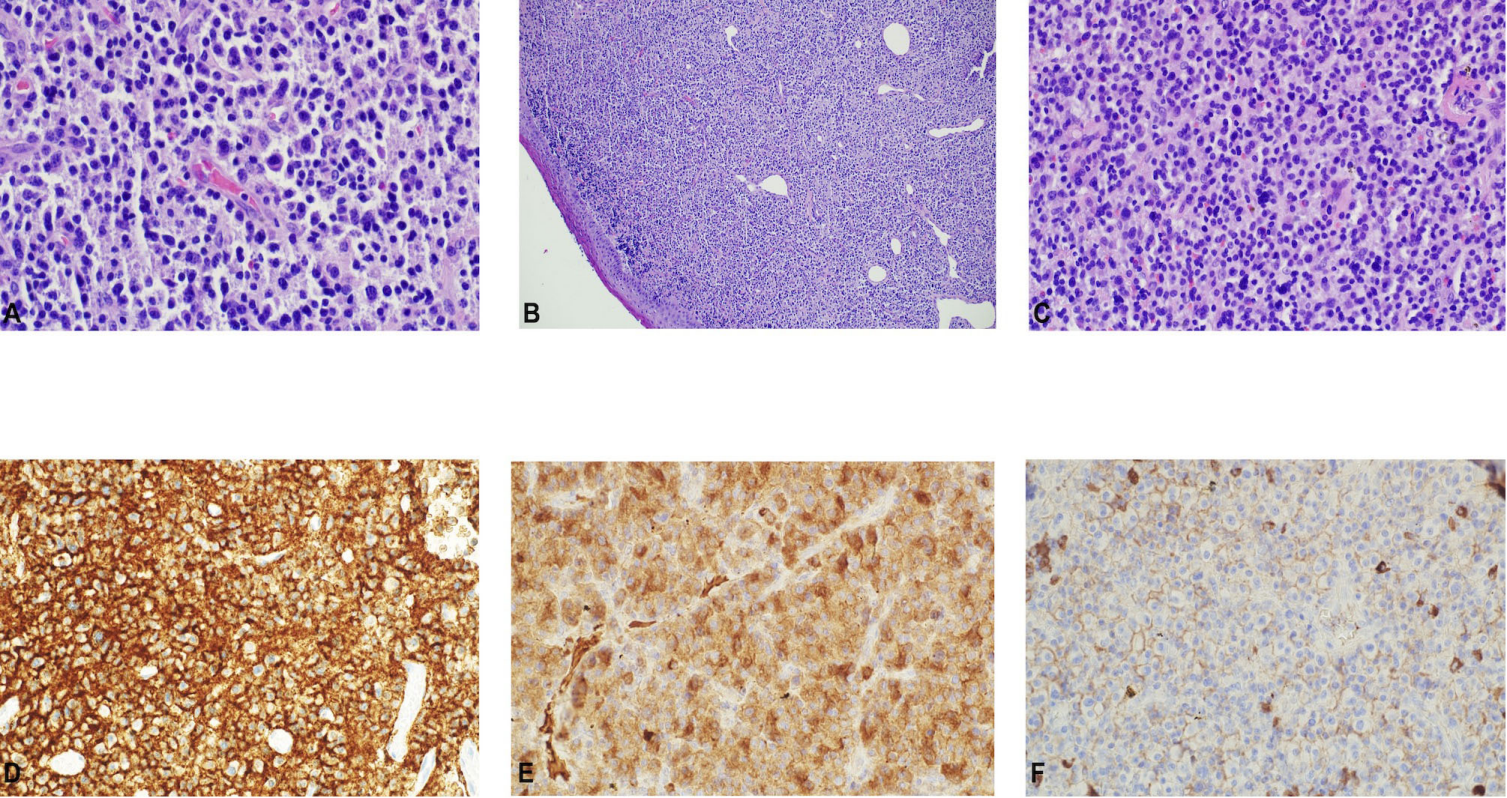
Histopathological assessment of the tumor. **(A–C)**. Throat tumor biopsy showing sheets of plasma cells. H&E. (x 400). Immunohistochemistry displaying positivity for **(D)** CD138, **(E)** lamda, and the **(F)** absence of kappa light chain expression.

**Figure 3:**
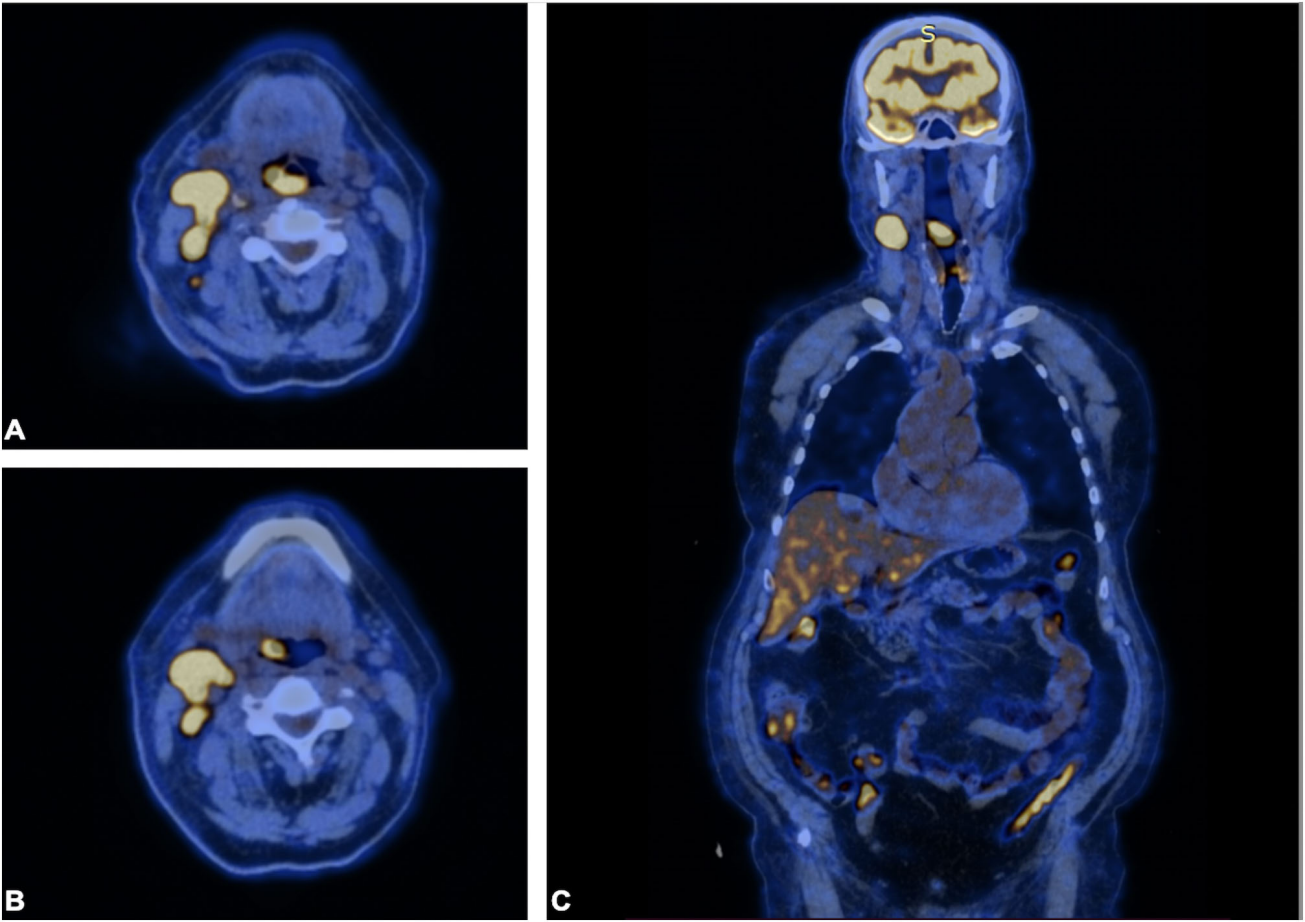
PET scan. PET scan showing increased FDG uptake in a **(A, B)** soft tissue density lesion in the right throat originating from the right piriform fossa (horizontal section) and in the **(C)** right cervical lymph node of IIA group (coronal section). Note that no abnormal FDG avid uptake was observed elsewhere, indicating the absence of a distant lesion.

**Figure 4:**
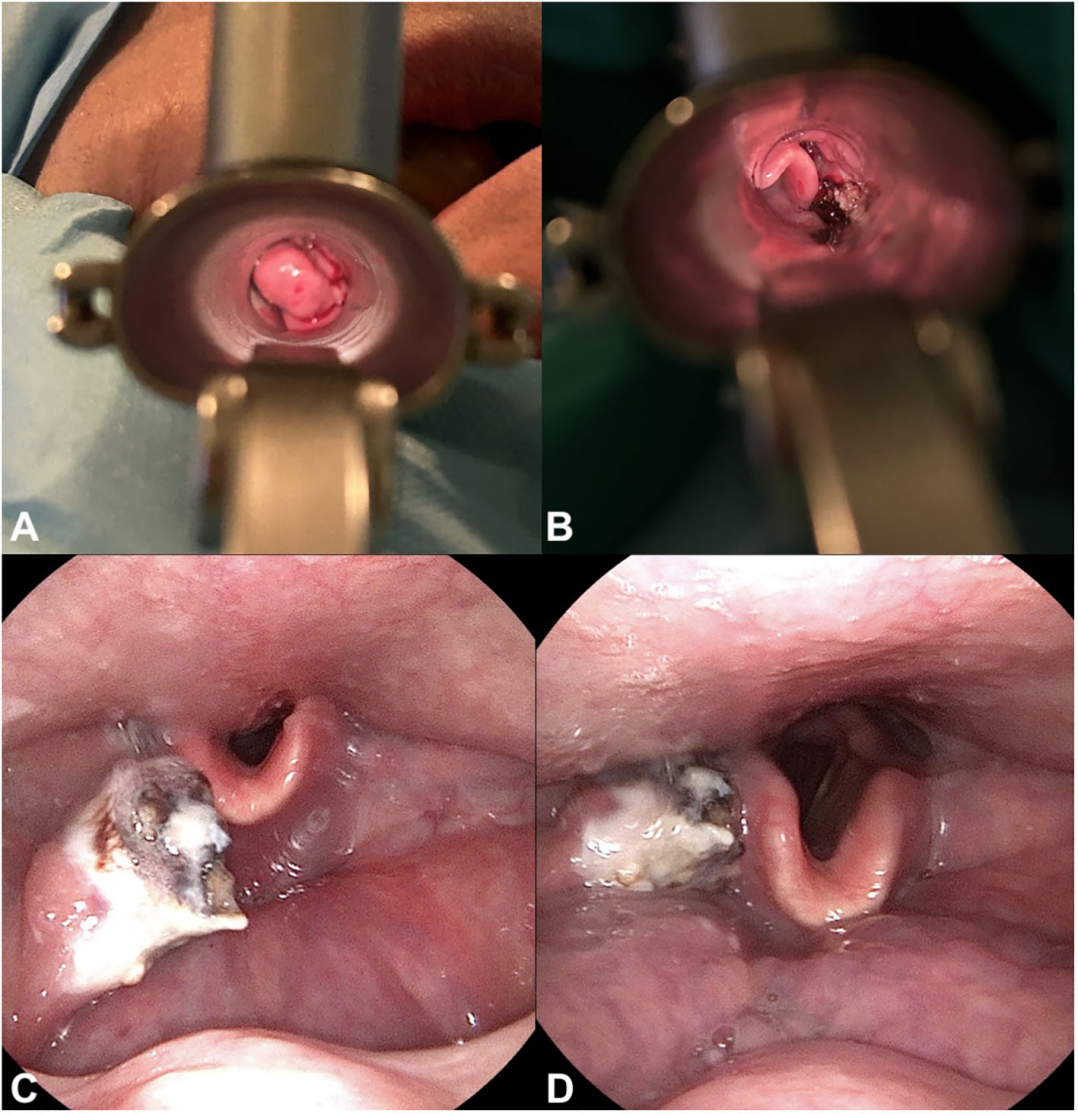
Surgical procedure and post-surgical fiberoptic exam. Visualization of the tumor through a laryngoscope, shown **(A)** during the surgical removal of the tumor and **(B)** post-removal. **(C, D).** Fiberoptic examination post-surgery revealing residual tumor tissue.

A 72-year-old male of Polish origin was admitted to the Head and Neck Cancer Clinic at the National Institute of Oncology in Poland with a 6-month history of swallowing problems. He denied weight loss but admitted occasional swallowing difficulties, with no reported hoarseness or pain on swallowing. His past medical history was notable for hypertension, and there was no family history of neoplastic, genetic, or metabolic disorders. The patient denied cigarette smoking or alcohol use disorder. On presentation, his vital signs were within normal limits (HR: 87, RR: 132/87 mm Hg, temperature: 36.6°C). Physical examination revealed no abnormalities in the mouth or nasal cavities, but an enlarged submandibular lymph node was palpated on the right side of the neck. Indirect laryngoscopy and fiberoptic exams revealed a round 2 cm, smooth polypoid mass protruding from the right piriform fossa, partially occluding the vocal slit during phonation (**Figures 1A, B**). Laboratory tests showed Hb 13 g/dL, calcium level of 10.5 mg/dL, free kappa chains of 18.30 mg/L, free lambda chains of 15.90 mg/L, with a Kappa/Lambda ratio of 1.15. Additionally, normal serum BUN/creatinine, sodium, potassium, albumin, and LDH levels were detected. The patient underwent a whole body CT scan, and based on the laboratory results, a hematological and oncological consultation was ordered to rule out lymphoma, multiple myeloma, or other hematological malignancies. Further tests included serum quantitative immunoglobulins (IgM: 42.3 mg/dL, IgG: 796 mg/dL, IgA: 245 mg/dL), serum protein electrophoresis, monoclonal protein immunofixation (all normal), and a slightly elevated beta-2-microglobulin level of about 3.33 mg/L. No Bence-Jones protein was detected in the 24-hour urine collection. The CT scan revealed a well-circumscribed polypoid homogeneously enhancing soft tissue mass adjacent to the posterior surface of the epiglottis and the right side of the tongue base, approximately measuring 17x11mm x 23mm. An enlarged lymph node posterior to the right submandibular gland, reaching about 23x20mm, was also noted. Subsequently, the patient underwent an open biopsy procedure of the tumor, along with a bone marrow biopsy. The tumor pathological findings confirmed the diagnosis of EMP (**Figures 2A–F**). Additionally, since the patient showed no signs of multiple myeloma, and the bone marrow biopsy yielded normal results, the diagnosis of multiple myeloma was excluded. Moreover, the histopathological findings helped rule out diagnoses such as lymphoma, sarcoma, or squamous cell carcinoma (SCC).Given the histopathological, laboratory, and imaging findings, the patient was scheduled for a re-consultation with a hematologic specialist and radiotherapist to initiate treatment. Subsequently, the patient underwent intensity-modulated radiation therapy (IMRT) with a total dose of 42 Gy over the next 4 weeks, comprising five sessions per week. Nevertheless, the RT turned out to be unsuccessful with no evidence of a tumor decrease on fiberoptic exam upon therapy completion. Due to the unsuccessful response to the radiotherapy, the patient underwent a PET/CT scan and was scheduled for surgical tumor removal. Whole-body PET/CT images displayed an increased FDG uptake in a soft tissue density lesion in the right throat originating from the right piriform fossa, as well as in the right cervical lymph node of IIA group, with no abnormal FDG avid uptake elsewhere suggesting a distant lesion. The maximum standardized uptake value of the tumor was 7.1 (**Figures 3A, B**). The surgical procedure was conducted under general anesthesia, with the use of a laryngoscope. The tumor was visualized and partially removed using microlaryngeal scissors and bipolar coagulation, as complete resection was not feasible, and only a debulking approach was applied (**Figures 4A, B**). Post-surgery, the patient underwent a follow-up visit after two weeks, during which a fiberoptic exam revealed a residual tumor mass (**Figures 4C, D**). Following this, the patient was referred for a hematological and oncological consultation to proceed with a chemotherapeutic regimen of dexamethasone and bortezomib, but due to severe side effects including numerous bleeding episodes, anemia and dizziness, and the absence of a MM diagnosis, chemotherapy was discontinued after one course. The therapeutic regimen comprised subcutaneous injections of bortezomib at a dose of 1.3 mg/m^2^ on days 1, 4, 8, and 11 of a 21-day treatment cycle, along with dexamethasone administered at 20 mg on the day of and the day following bortezomib injection. Regular follow-up included fiberoptic exams every 3 months during the first year, MRI every 6 months, and a PET scan once per year. It has been 18 months since the diagnosis of EMP, and the patient remains under observation at the National Institute of Oncology with no signs of multiple myeloma or tumor recurrence detected thus far. Despite initially feeling overwhelmed by the diagnosis and the lack of response to radiotherapy, the patient remained calm and optimistic about recovery. Eventually, his convalescence was uneventful, with no tumor recurrence observed after the surgical and chemotherapeutic approach.

## Discussion

In this case scenario, we present a rare example of EMP without a concomitant diagnosis of multiple myeloma. EMP is an uncommon malignancy originating from plasma cells and can manifest in soft tissues throughout the body. Typically, EMP is characterized by a slow-growing submucosal tumor within the respiratory and digestive tract, with the head and neck region being the most common location (6, 10). Predominantly observed in nasal cavities, paranasal sinuses, nasopharynx, oropharynx, and larynx, EMP’s symptoms are location-dependent and primarily associated with the mass effect, leading to issues such as swallowing difficulties, breathing problems, epistaxis, or nasal obstruction (6, 11). EMP does not affect the bone marrow but can progress to multiple myeloma (MM) (12). Two forms of EMP are recognized: primary and secondary. Primary EMP denotes tumors present without any other plasma cell malignancies, such as multiple myeloma or monoclonal gammopathy. On the other hand, secondary EMP occurs in the presence of other plasma cell dyscrasias, most commonly MM (8). To date, numerous cases of EMP without MM have been reported in the literature, with several cases presenting the tumor in the laryngopharynx location (13–17). Given its rarity, a high index of suspicion is required for an accurate diagnosis, necessitating tumor and bone marrow biopsies to assess the level of monoclonal plasma cells (7). Histopathological examination of the tumor tissue reveals a dense infiltrate of plasmablastic or anaplastic plasma cells, which may be immature or mature (18). However, the similarities in histopathological features and the high pleomorphism of plasma cells may lead to confusion with aggressive lymphoma, rhabdomyosarcoma, or other sarcomas (19). Specifically, EMP should be distinguished from plasmablastic lymphoma, which exhibits overlapping features with myeloma and lymphoma. Plasmablastic lymphoma comprises B-cells, lymphoplasmablastic cells, and monoclonal cells with an immunohistochemical profile that typically includes CD20 expression, but not CD138 and is often associated with Epstein-Barr virus (EBV) infection (20). It is a highly aggressive lymphoma observed in immunocompromised patients, primarily affecting the oral cavity and gastrointestinal tract (17). Conversely, EMP may also be occasionally confused with angiosarcoma. However, angiosarcoma, consisting of atypical endothelial cells that may resemble plasmocytes, typically induces the destruction of surrounding tissues (21). Additionally, an extremely rare anaplastic MM may occasionally mimic EMP. Originating from immature plasma cells and presenting with extramedullary tumors, it leads to bone marrow invasion and osseous lytic lesions, which are not observed in EMP (22). Another condition that can sometimes mimic EMP is SCC, which is the most common malignancy in the head and neck region (23). Despite the similarity in location, EMP typically presents with localized growth, while SCC is more aggressive with a higher potential for metastasis. Additionally, poorly differentiated SCC cells may sometimes test positive for CD138 or plasma cell markers, making it crucial to use a comprehensive panel of antibodies to differentiate between carcinoma and EMP (24). On the other hand, large B-cell lymphoma (LBCL) cells, while they may share similar microscopic characteristics with EMP, typically express CD19, CD20, CD79a, and PAX5, but are negative for plasma cell markers such as CD138 and CD38 (25). Additionally, LBCL typically presents as a rapidly growing mass with extranodal involvement in over 50% of cases with stomach or gastrointestinal tract most commonly affected (26).

Furthermore, for adequate locoregional staging of EMP, a CT scan or MRI is essential. However, a PET scan appears to be the preferred imaging study for ruling out MM or other hematologic malignancies. The diagnostic approach should encompass the following evaluation criteria: (i) biopsy of a solitary lesion of bone or soft tissue with evidence of clonal plasma cells, (ii) bone marrow biopsy within normal limits with no clonal plasma cells, (iii) no abnormalities in the skeletal survey displayed on MRI (or CT) of the spine and pelvis (except for the primary solitary lesion), (iv) no evidence of hypercalcemia, anemia, renal insufficiency, or bone lesions (CRAB) caused by a lymphoplasmacytic cell proliferative disorder (27).The rarity of EMP and its prolonged course make it challenging to determine the optimal treatment strategy. In the presented case study a radiotherapeutic regimen was used as a primary approach as it is the preferred first-line treatment of EMP due to the tumor’s radiosensitivity (28). This approach is considered feasible, particularly when the location of the EMP presents challenges for surgical intervention, such as proximity to vital and sensory organs. According to the literature, RT is typically administered over a 4-week period with a total dose ranging from 40 to 50 Gy (29). In the majority of EMP cases, radiotherapy is effective, demonstrating excellent disease control and a low risk of recurrence (30). Given the radiosensitive nature of EMP, RTH remains the treatment of choice. However, in cases refractory to radiotherapy, other therapeutic approaches need to be employed. Depending on the tumor location and the proximity of critical structures, radical surgical resection may not be feasible. Therefore, additional treatment modalities are required. In a case report described by Karakullukcu et al., surgical debulking combined with photodynamic therapy was employed to manage residual EMP tumor resulting in a successful outcome (31). Another therapeutic approach presented in the available literature involved bronchoscopic electrocautery snare combined with CO2 cryotherapy, followed by RTH for EMP located in the trachea (32). In the presented case report, a surgical debulking approach was employed because radical resection was not feasible. Consequently, due to the risk of progression to MM, lack of response to RTH and the residual tumor, the patient underwent a subsequent chemotherapeutic regimen of dexamethasone and bortezomib. Nevertheless, existing literature suggests that the majority of cases of EMP respond well to radiotherapy, and the other treatment options should be implemented in case of radiotherapy resistance. The current publications confirm positive outcomes with surgery and/or systemic therapy, including bortezomib or cyclophosphamide alone or in combination with vincristine and prednisolone (33). In their case report on gastric EMP, Katodritou et al. were the first to present dexamethasone and bortezomib as an effective treatment approach for managing EMP without associated multiple myeloma (34). Similarly, in a case report by Wei et al., pancreatic EMP was successfully treated with a combination of bortezomib and dexamethasone after the tumor failed to respond to the traditional chemotherapy regimen of vincristine, doxorubicin, and dexamethasone (the VAD regimen) (35). Moreover, certain immunomodulatory agents, such as daratumumab, have also been found to be effective in EMP patients (36). However, the potential toxicity of chemotherapy, including risks of myelosuppression, neuropathy, and organ toxicity, are significant drawbacks that can limit its application. Decisions regarding chemotherapy should consider factors like the patient’s overall health, existing comorbidities, potential drug interactions, and the severity or progression of the disease. In our patient, poor tolerance to chemotherapy and the absence of signs of MM led to the discontinuation of the chemotherapy regimen after just one course. Moreover, it appears that the resistance to radiotherapy in EMP tumors requires further molecular research to assess the likelihood of the radiotherapy efficacy. As such, this rare case study of EMP without multiple myeloma diagnosis proves the rarity of this malignancy as well as shed some light on its possible resistance to radiotherapy. Although the patient described in this manuscript achieved recovery with no evidence of tumor recurrence, he experienced side effects of initial radiotherapy, including xerostomia and radiation-induced skin reactions. A significant limitation of this case report is the inability to generalize these findings and to establish causality. However, despite these limitations, this case report highlights a rare and unusual case of EMP that is refractory to radiotherapy, underscoring the importance of a personalized medicine approach and raising awareness among healthcare professionals about EMP. Though, the presented case underscores the need for further research on a larger group of patients with EMP to identify the most effective therapeutic approach. This case report underscores the exceptionally rare form of EMP without MM, further compounded by its resistance to radiotherapy, rendering it an even more unique case within the EMP spectrum. A high degree of clinical suspicion is necessary for diagnosing EMP. Namely, counseling patients with EMP demands a considerate and patient-centered approach, considering the rarity of the condition and its potential for progression to MM. While SCC remains the most common malignancy within the head and neck region, the diagnosis of EMP should always be considered in a patient with a tumor in this region. Consequently, there is an imperative for clinicians to maintain a high index of suspicion when assessing EMP patients and determining the most appropriate treatment plan.

## Data availability statement

The raw data supporting the conclusions of this article will be made available by the authors, without undue reservation.

## Ethics statement

Written informed consent was obtained from the individual(s) for the publication of any potentially identifiable images or data included in this article. Written informed consent was obtained from the participant/patient(s) for the publication of this case report.

## Author contributions

KS: Writing – review & editing. AGa: Writing – review & editing, Investigation. AGo: Writing – review & editing, Supervision. MD: Writing – review & editing, Supervision, Investigation. KB-P: Writing – review & editing, Investigation. JZ: Writing – review & editing, Supervision.
