# Supplementary material for: Case report: An uncommon presentation of extramedullary plasmacytoma without a concurrent diagnosis of multiple myeloma
Source: Front Oncol. 2024 Jun 7;14:1353943. doi: 10.3389/fonc.2024.1353943 (PMC11190079; doi:10.3389/fonc.2024.1353943)
Supplement: Supplementary file 1 [file DataSheet_1.pdf]

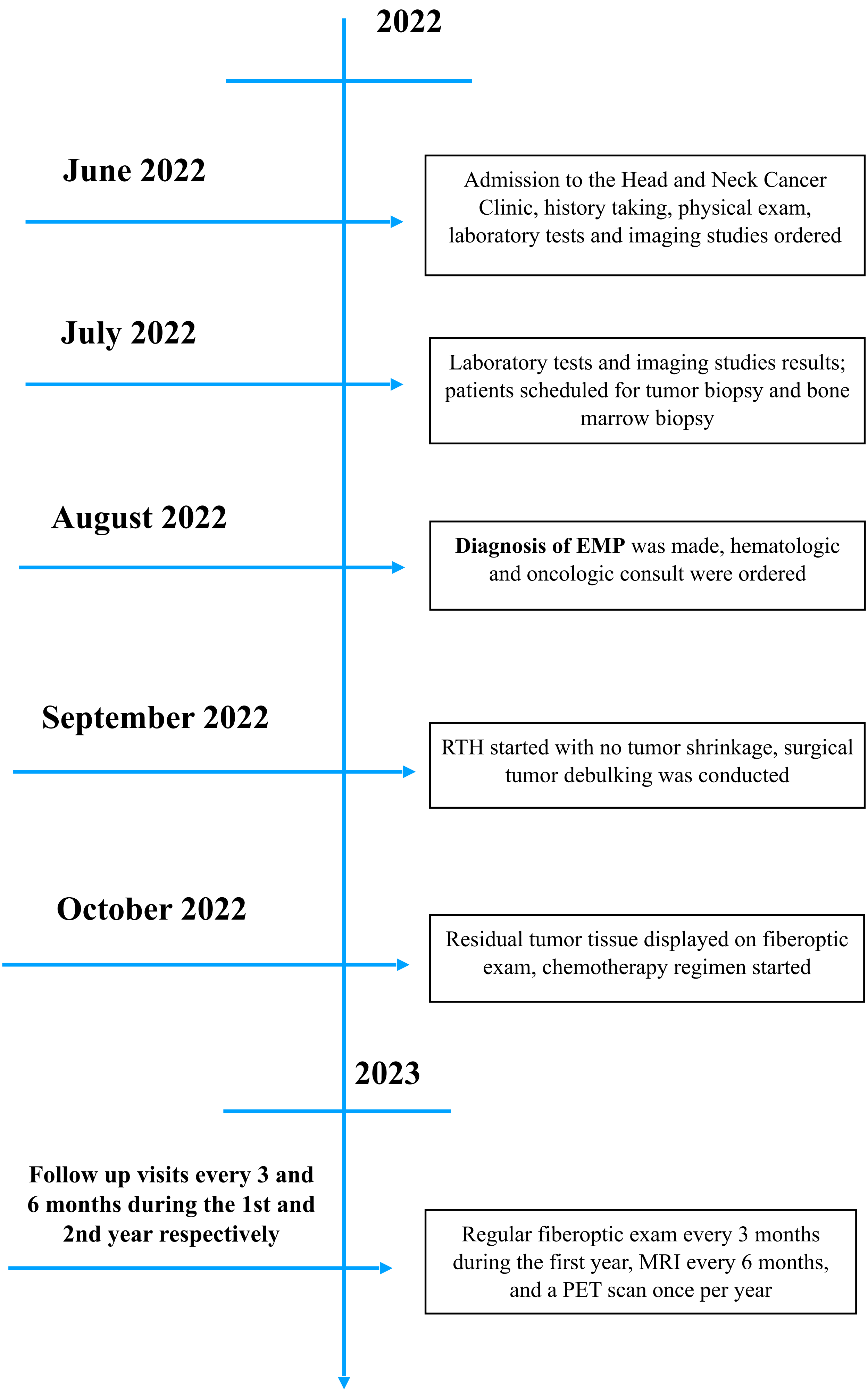
2022
June 2022
Admission to the Head and Neck Cancer Clinic, history taking, physical exam, laboratory tests and imaging studies ordered
July 2022
Laboratory tests and imaging studies results; patients scheduled for tumor biopsy and bone marrow biopsy
August 2022
Diagnosis of EMP was made, hematologic and oncologic consult were ordered
September 2022
RTH started with no tumor shrinkage, surgical tumor debulking was conducted
October 2022
Residual tumor tissue displayed on fiberoptic exam, chemotherapy regimen started
2023
Follow up visits every 3 and 6 months during the 1st and 2nd year respectively
Regular fiberoptic exam every 3 months during the first year, MRI every 6 months, and a PET scan once per year
